# Mitochondrial Bioenergetics in Different Pathophysiological Conditions

**DOI:** 10.3390/ijms22147562

**Published:** 2021-07-15

**Authors:** Daniela Valenti, Anna Atlante

**Affiliations:** Institute of Biomembranes, Bioenergetics and Molecular Biotechnologies (IBIOM)-CNR, Via G. Amendola122/O, 70126 Bari, Italy

Mitochondria are complex intracellular organelles involved in many aspects of cellular life, with a primary role in bioenergy production via oxidative phosphorylation (OXPHOS).

In this Special Issue, nine review papers were published dealing with compelling topics related to mitochondrial bioenergetics and other linked multifunctional roles of mitochondria in various pathophysiological contexts.

Mitochondrial OXPHOS disorders are critical contributors involved in the pathogenesis of a broad range of human diseases directly or indirectly through a wide spectrum of signaling pathways [1]. In their review, Protasoni and Zeviani have analyzed, in detail, the structure, the assembly pathway and the organization in supercomplexes of selective components of the OXPHOS machinery, reviewing how single or isolated defects in specific mitochondrial respiratory chain proteins are linked to a variety of human disorders [2]. The authors discussed the heterogeneity and the complexity behind the still-growing group of pathologies normally identified as “mitochondrial diseases”, the pathophysiology of which is still often poorly understood. Among them, primary mitochondrial diseases are associated with genetic mutations both in nuclear and mitochondrial DNA (mtDNA), affecting genes involved in every aspect of the organelle function [2].

In the energy metabolism, the mitochondrial adenine nucleotide translocator (ANT) plays the fundamental role of gatekeeper and key regulator of cellular bioenergetic flow, carrying out the reversible exchange of ADP for ATP across the inner mitochondrial membrane [3]. In their review, Atlante and Valenti focused on this mitochondrial translocator, making “*A walk in the memory: from the first Functional Approach up to Its Regulatory Role of Mitochondrial Bioenergetic Flow in Health and Disease*” [3]. In particular, the authors reviewed and discussed among their studies all those in which they were able to measure the functional activity of ANT in a variety of pathophysiological contests, by using an—ancient but still actual- experimental strategy allowing them to monitor continuously the transfer of energy in the form of ATP from the inside of the mitochondria to the outside, under conditions close to a biological status reflecting what really happens in the cellular microenvironment [3].

Mitochondria are highly dynamic organelles, the morphology of which is tightly linked to their functions, ongoing fusion and fission events, reshaping their morphology as well as to relocate within the cell, thus supporting critical energy power needs [4]. In addition, growing evidence established a connection between dysregulated mitochondrial dynamics and disease development and progression. In particular, defects in key components of the machinery mediating mitochondrial dynamics have shown to be linked with a wide range of pathological conditions, including metabolic, immune and neurodegenerative diseases [5]. Regarding this, a further update on the molecular mechanisms promoting mitochondrial fusion and fission in mammals has been provided by Kyriakoudi et al. [6]. The authors discussed on the emerging association of human diseases with aberrant mitochondrial dynamics, making the latter as a major culprit for disease pathogenesis and an attractive target for therapy.

Neurological disorders, including neurodegenerative diseases, are collectively a major cause of death and disability worldwide. Altered mitochondrial function has been implicated in most of these diseases, but key information about how this dysfunction occurs, whether it is a cause or an effect, how it differs in different neurological conditions and how the dysfunction progresses, is lacking. Moreover, studying mitochondrial function in these disorders is difficult due to the inaccessibility of brain tissue, which is the primary tissue affected in these diseases. Thus, to overcome this issue, numerous cell models have been used, each providing unique benefits and limitations. In their compelling study, Annesley and Fisher focused on the use of lymphoblastoid cell lines (LCLs) to study mitochondrial function in neurological disorders [7]. These cell models have long been used as tools for genomic analyses, but in their article the authors reviewed the functional studies using LCLs, in specifically as concerns mitochondrial function [7]. LCLs have enabled characterization of the underlying mitochondrial defect, identification of altered signaling pathways and proteins, differences in mitochondrial function between subsets of particular disorders and identification of biomarkers of the disease. The examples provided in their review suggest that these cells could be useful for development of diagnostic tests, identification of drug targets and testing of pharmacological agents, constituting a valuable model for studying mitochondrial function in neurological disorders.

Evidence shows that after the nerve injury, mitochondrial bioenergetic dysfunction occurs and is associated with pain, neuropathy and nerve regeneration deficit. A challenge for research is to individuate new therapies able to normalize mitochondrial and energetic metabolism to aid nerve recovery after damage. Regarding this, Ravera et al. discussed the reliability of photo-biomodulatory event strongly based on biological and physical–chemical evidence, reviewing as mitochondria are principal players of photomodulation, whether their cytochromes are directly involved as photo-acceptor or indirectly through a vibrational and energetic variation of bound water and water as a photo-acceptor [8,9]. Moving from a microscopic point of view to preclinical and clinical studies, the authors reviewed as photo-biomodulation appears to play an effective medical support for trigeminal nerve disease [10].

Another issue examined in the context of mitochondrial bioenergetics concerns the crucial role played by mitochondria in the regulation of muscle mass and quality via retrograde signaling to the nucleus, involving oxygen radical production, energy deficits and apoptosis. Therefore, maintenance of the organelle reticulum is critical to either offset or minimize the detrimental outcomes of muscle chronic disuse. Periods of muscle disuse promote marked mitochondrial alterations that contribute to the impaired metabolic health and degree of atrophy in the muscle. Thus, understanding the molecular underpinnings of muscle mitochondrial decline with prolonged inactivity is of considerable interest. In their review, Memme et al. addressed this topic discussing the “*Mitochondrial Bioenergetics and Turnover during Chronic Muscle Disuse*” [10].

The therapeutic effects of exercise have been observed to mitigate and/or reverse the disuse-induced maladaptive phenotype by restoring the signaling events that initiate mitochondrial biogenesis and proper organelle clearance, while also abolishing the harmful ROS levels via restoration of mitochondrial antioxidants. The evidence reveals that sustained muscle contractions, as with aerobic exercise, greatly improve the mitochondrial derangements that develop during periods of inactivity [10]. Rehabilitative training following periods of inactivity is sufficient to re-establish mitochondrial volume and function within the muscle, thus restoring muscle mass, strength and endurance back toward normal levels. Thus, the beneficial outcomes of contractile activity provide optimism in improving muscle health and the quality of life of those subjected to extended periods of inactivity. Future work in this area should include further delineation of the mechanisms connecting disuse-induced mitochondrial dysfunction and the expression of genes controlling muscle wasting, and specifically which potential molecular targets can be exploited to regulate mitochondrial health in the face of atrophic conditions.

The study of necroptosis addressed by Chen et al. is particularly captivating [11]. Necroptosis is a common form of programmed cell death in the liver. The necrosome complex RIPK1/RIPK3/MLKL (composed by receptor-interactive protein kinase 1/receptor -interactive protein kinase 3/ mixed lineage kinase domain-like protein), was proposed to induce necroptosis via mitochondrial dysfunction involving a variety of mechanisms, including induction of mitophagy and production of mitochondrial reactive oxygen radicals (reviewed in [11]). In their review, Chen et al. focused on the role of mitochondria-mediated cell necroptosis in acute liver injury, chronic liver diseases and hepatocellular carcinoma, and the possible translation of these processes into clinical applications [11].

Recent evidence suggests that epigenetic modifications of the mitochondrial genome could also contribute to the etiology of human diseases. In their review, Stoccoro and Coppodè discussed the role of mitoepigenetics in the regulation of mitochondrial 3metabolism, highlighting the role of DNA methylation inside this organelle and its impact on human diseases. They also debated the possible impact of the nuclear genetic background and of environmental factors in the regulation of mtDNA methylation [12].

Chinopoulos’ review is particularly engaging [13]; this deals with the lysine succinylation process. Lysine succinylation is a post-translational modification which alters protein function in both physiological and pathological processes. Aware that it requires succinyl-CoA, a metabolite formed within the mitochondrial matrix that cannot permeate the inner mitochondrial membrane, the question arises as to how there can be succinylation of proteins outside mitochondria. In this review, Chinopoulos examined pathways participating in peroxisomal fatty acid oxidation leading to succinyl-CoA production, potentially supporting the succinylation of extramitochondrial proteins [13]. Furthermore, since some of those pathways depend on NAD^+^ availability, the connection of cytosolic protein lysines succinylation to mitochondrial status is examined. Finally, the discovery that glia in the adult human brain lack of subunits of both alpha-ketoglutarate dehydrogenase complex and succinate-CoA ligase—thus being unable to produce succinyl-CoA in the matrix—and yet exhibit robust pancellular lysine succinylation, is highlighted [13].

It is a pleasure for the Guest Editors to gratefully acknowledge all the authors for the quality of their contributions into this Special Issue. Last but not least, the Editors wish to extend special thanks to the Assistant Editor Shanny Li for her constant and valuable support and the members of the editorial staff of the Multidisciplinary Digital Publishing Institute (MDPI) for their professionalism. Hopefully, readers will enjoy this Special Issue and be inspired by new ideas for future researches.

With great joy, we take this opportunity to communicate that, given the great success, the Special Issue “*Mitochondrial Bioenergetics in Different Pathophysiological Conditions*” has launched the Second Volume. It is open to all those who have not been able to contribute with an original or review study to the second volume. Here the link: https://www.mdpi.com/journal/ijms/special_issues/Mitochondrial_Bioenergetics_2 (accessible until 30 November 2021).

## Data Availability

Not applicable.
